# Supplementary material for: Patterns of island change and persistence offer alternate adaptation pathways for atoll nations
Source: Nat Commun. 2018 Feb 9;9:605. doi: 10.1038/s41467-018-02954-1 (PMC5807422; doi:10.1038/s41467-018-02954-1)
Supplement: Supplementary file 1 — Supplementary Information [file 41467_2018_2954_MOESM1_ESM.docx]

**Supplementary Note 1**

**Tuvalu Archipelago Form and Structure**

The Tuvalu archipelago is located in the tropical south Pacific and extends 680 km from Nanumea (5^o^ 38.7’S, 176 ^o^ 3.9’ E) in the northwest to Niulakita (10^o^ 47.352’S, 179 ^o^ 28.4’ E) in the southeast (Supplementary Figure 1, Supplementary Table 1). The archipelago comprises five atolls and four reef platforms. There are a total of 101 vegetated islands with an aggregated land area of approximately 2,552 hectares. However, the distribution of islands and land area is not evenly distributed. Atolls comprise an outer coral reef rim that encircle distinct lagoons, which range in depth up to 80 m (Supplementary Figure 2). Ninety islands are found on the peripheral rim of atolls in Tuvalu (Supplementary Figure 2), which have a total land area of 1,451 hectares, approximately 57% of land area in the archipelago. Reef platforms are structurally distinct from atolls as they are smaller tabular reef platforms that have no marked lagoon (Supplementary Figure 2). Islands occupy the central portion of the reef platform surface. The four reef platform islands in Tuvalu contain a total of 11 islands (although four dominate), have total land area of 1,101 ha, approximately 43.1% of total land area in the archipelago.


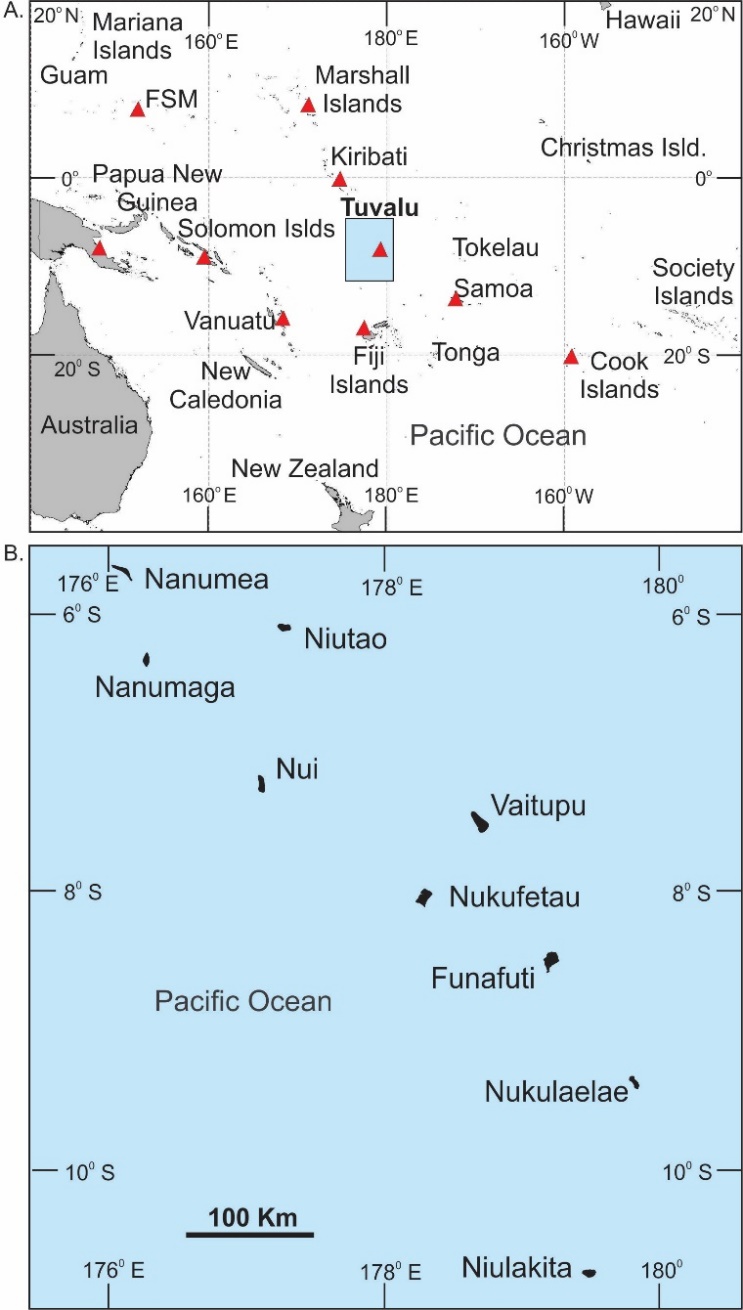


**Supplementary Figure 1.** The location of the Tuvalu archipelago, South Pacific Ocean (A) and location of the nine atolls and reef platforms (B).

**Supplementary Table 1.** Location, structure and size of the reefs and reef platforms of Tuvalu.

| **Name** | **Reef Type** | **Latitude** | **Longitude** | **Total Area (km^2^)** |
| --- | --- | --- | --- | --- |
| Nanumea | Atoll | 5^o^ 38.702’ S | 176^o^ 3.925’ E | 22.0 |
| Niutao | Reef Platform | 6^o^ 6.552’ S | 177^o^ 20.494’ E | 2.5 |
| Nanumaga | Reef Platform | 6^o^ 17.232’ S | 176^o^ 19.214’ E | 3.0 |
| Nui | Atoll | 7^o^ 13.470’ S | 177^o^ 9.206’ E | 17.0 |
| Vaitupu | Reef Platform | 7^o^ 28.864’ S | 178^o^ 40.782’ E | 10.0 |
| Nukufetau | Atoll | 7^o^ 59.638’ S | 178^o^ 22.500’ E | 145.0 |
| Funafuti | Atoll | 8^o^ 32.195’ S | 179^o^ 6.776’ E | 277.0 |
| Nukulaelae | Atoll | 9^o^ 23.484’ S | 179^o^ 50.443’ E | 43.0 |
| Niulakita | Reef Platform | 10^o^ 47.352’ S | 179^o^ 28.404’ E | 0.5 |


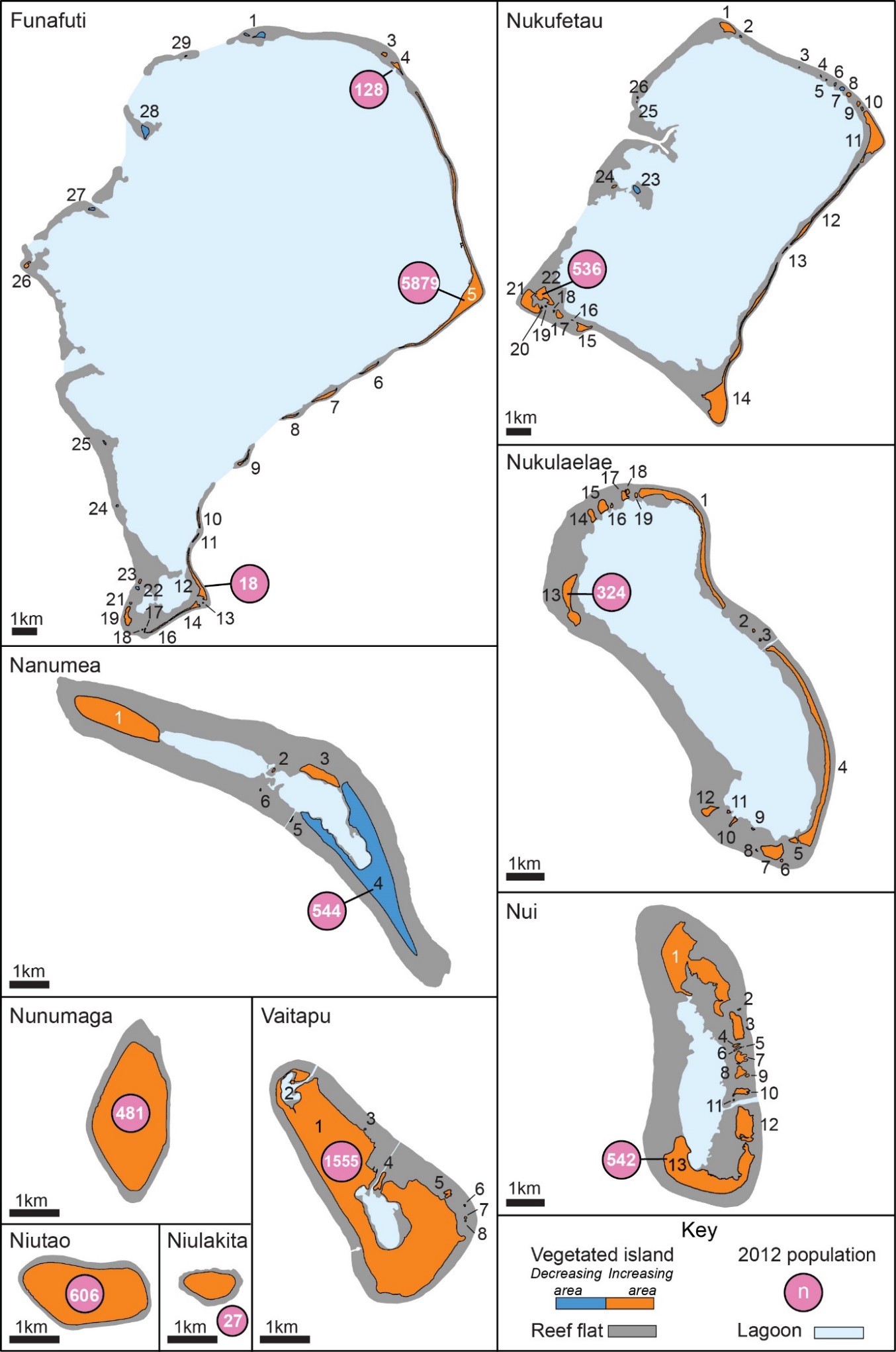


**Supplementary Figure 2.** Planform characteristics of the atolls and reef platforms of the Tuvalu archipelago. Numbers denote sequence of islands analysed for each atoll or reef platform. Population values of inhabited islands are shown in circles.

**Supplementary Note 2**

**Funafuti Sea Level Record**

Sea level has been measured nearly continuously at Funafuti atoll since November 1977. Monthly water level data is provided by the Permanent Service for Mean Sea Level (PSMSL) <http://www.psmsl.org/>. The monthly record is comprised of observations made at two at locations within Funafuti lagoon. From November 1979 until December 2001 the University of Hawai’i Sea Level Center (UHSLC) operated a tide gauge on a wharf within the lagoon (Funafuti-A). Since June 1993 the National Tidal Centre of the Australian Bureau of Meteorology has operated a tide gauge within the lagoon ~2.5 km from the UHSLC tide gauge (Funafuti-B). The two records were synthesised into a single 1977-2015 time series by averaging the difference between the A and B records over the period during which both gauges operated simultaneously. The Funafuti-B record was then reduced using the offset to provide a near-continuous record of water level at Funafuti (Supplementary Figure 3).

The sea level record from Funafuti atoll between 1977 and December 2015 reveals significant interannual variability and an annual rate of sea level rise was 3.9 ± 0.4 mm yr^-1^. Across the timeframe of analysis this rate of sea level change equates to a ~0.15 m over the past 40 years (~0.39 m per century). The data shows that islands in Tuvalu have been subject to rates of sea level change approximately twice the global average over the past four decades^48^.

**
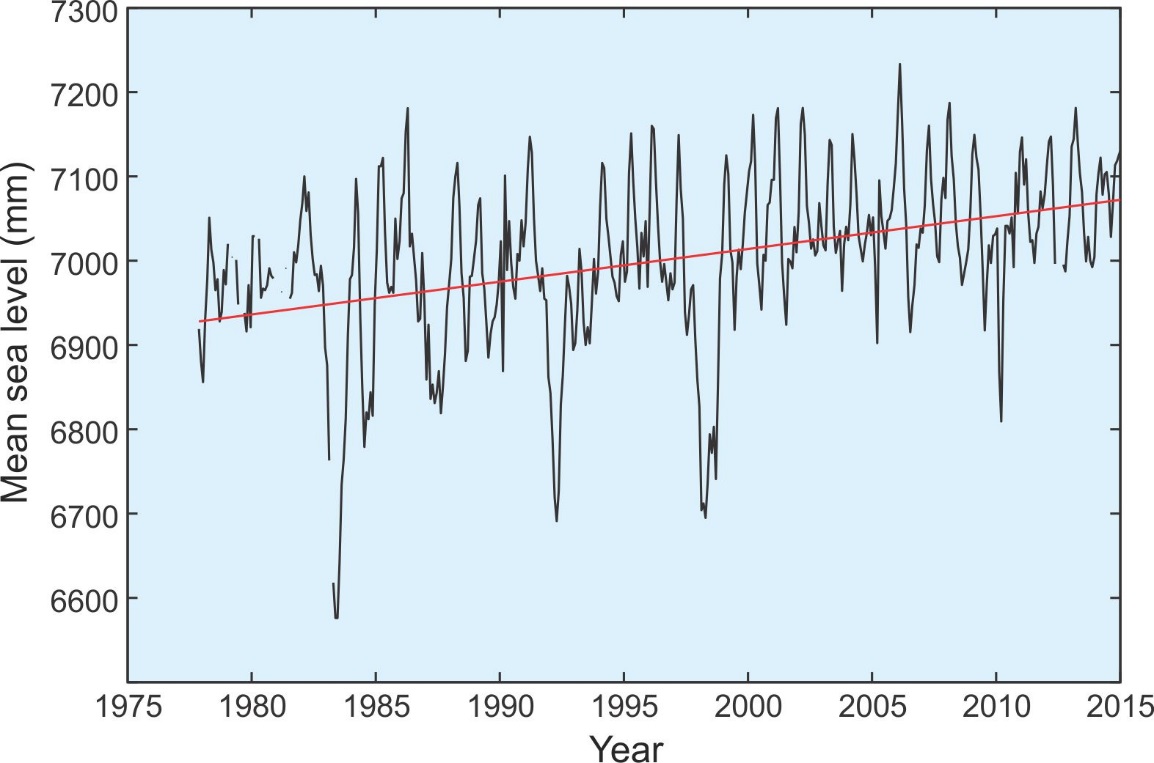
**

**Supplementary Figure 3.** Funafuti sea level record 1977-2015. Source: Permanent Service for Mean Sea Level – <http://www.psmsl.org>
