# Supplementary material for: Patterns of island change and persistence offer alternate adaptation pathways for atoll nations
Source: Nat Commun. 2018 Feb 9;9:605. doi: 10.1038/s41467-018-02954-1 (PMC5807422; doi:10.1038/s41467-018-02954-1)
Supplement: Supplementary file 3 — Description of Additional Supplementary Information [file 41467_2018_2954_MOESM3_ESM.docx]

**Description of Additional Supplementary Files**

File Name: Supplementary Data 1

Description: Summary data of island area and island change in Tuvalu.

File Name: Supplementary Data 2

Description: Summary DSAS analysis of transects change on each island, Tuvalu.
